# Current knowledge of leptin in wound healing: A collaborative review

**DOI:** 10.3389/fphar.2022.968142

**Published:** 2022-09-12

**Authors:** Chi Yuan, Jian Liao, Liying Zheng, Lingzhi Ding, Xiao Teng, Xuesong Lin, Le Wang

**Affiliations:** ^1^ Department of Orthopedics, Taizhou Central Hospital (Taizhou University Hospital), Taizhou, Zhejiang, China; ^2^ Department of Nephrology, Jiaxing Hospital of Traditional Chinese Medicine, Jiaxing, Zhejiang, China; ^3^ Postgraduate Department, First Affiliated Hospital of Gannan Medical College, Ganzhou, China; ^4^ Department of Burn Surgery, Taizhou Central Hospital (Taizhou University Hospital), Taizhou, Zhejiang, China

**Keywords:** leptin, wound healing, inflammation, angiogenesis, re-epithelialization

## Abstract

Efficacious wound healing is still a major concern for global healthcare due to the unsatisfactory outcomes under the current treatments. Leptin, an adipocyte-derived hormone, mainly acts in the hypothalamus and plays crucial roles in various biological processes. Recently, an increasing number of researches have shown that leptin played an important role in the wound healing process. In this review, we presented a first attempt to capture the current knowledge on the association between leptin and wound healing. After a comprehensive review, the molecular mechanisms underlying leptin in wound healing were speculated to be correlated to the regulation of inflammation of the macrophage and lymphocytes, angiogenesis, re-epithelialization, proliferation, and differentiation of fibroblasts. The affected genes and the signal pathways were multiple. For example, leptin was reported to ameliorate wound healing by its anti-inflammatory action, which might be correlated to the activation STAT1 and STAT3 *via* p38 MAPK or JAK2. However, the understanding of the specific role in each process (e.g., inflammatory, proliferative, and maturation phase) of wound repair is not entirely clear, and further studies are still warranted in both macrostructural and microscale factors. Therefore, identifying and validating the biological mechanisms of leptin in wound healing is of great significance to develop potential therapeutic targets for the treatment of wound healing in clinical practice.

## Introduction

Millions of individuals suffer from skin injuries annually. Human skin wound healing is commonly accompanied by scar formation. Mild skin lesions categorized in wound healing can reach a complete regeneration, while moderate and severe injuries are usually associated with pathological tissue repairing and thus result in hypertrophic and keloid scars. For example, deep open wounds often cause abnormal healing cascades, dysregulating the sequence of inflammatory and regenerative cascades. Presently, efficacious wound healing is still a major concern for global healthcare due to the unsatisfactory outcomes under the current treatments, e.g., skin substitutes and alternative therapeutics (Heydari et al., 2022). In this regard, a better understanding of the molecular mechanisms of wound healing may be in favor of exploring effective curative interventions. A proper wound healing process is critical for skin repairs itself after injury (Ruiz-Canada et al., 2021). Wound healing is a vital physiological process consisting of three overlapping phases: an inflammatory phase, a proliferative phase, and a maturation phase, which involves angiogenesis, biosynthesis of the extracellular matrix (ECM), epithelialization, and tissue remodeling. Pathological wound healing is commonly caused by either superabundant scar tissue or failure to heal, which may lead to keloid scars, adhesions, chronic wounds, and surgical complications (i.e., anastomotic dehiscence and anastomotic leaks) (Zaharie et al., 2022). The common risk factors for pathological wound healing include infection, old aging, diabetes, and obesity. It is urgent to explore effective treatment methods for subjects with wound healing, especially in those with a high risk of difficulty healing. As a result, how to better control the wound healing process has important clinical relevance and implications.

Leptin is a hunger-sensing peptide hormone, secreted by adipose tissue. Several previous studies have unveiled that leptin, the product of the obese (ob) gene, influenced not only body weight homeostasis, but also hematopoiesis, reproduction, lipid metabolism, angiogenesis, and interaction with the immune system. Currently, leptin has also been shown to have an essential role in the wound healing process. However, the specific mechanism of leptin is controversial in wound healing. For instance, one study reported that leptin promoted wound healing by driving angiogenesis, while another study showed that leptin could not stimulate angiogenesis (Ring et al., 2000; Garonna et al., 2011). This article aims to summarize the recent advances in leptin for inflammatory response, angiogenesis, and epithelial regeneration.

## Overview of leptin

Leptin, the ob gene product, is a 16 kDa hormone predominantly expressed and secreted by mature adipose tissue and is encoded by the leptin gene located on chromosome 7q31.3. It is an α-helix type protein and belongs to the long-chain helical cytokine family, which includes leukemia inhibitory factor, human growth hormone, and ciliary neurotrophic factor. Leptin is mainly induced by adipose cells and enterocytes in the small intestine, thus yielding to regulate energy balance. Leptin was discovered in obese mice for the first time in 1994. Shortly after, the leptin gene was also reported in rats and humans. Many scholars believed that leptin gene expression in subcutaneous fat is lower than that in visceral fat, and its highest expression is usually in the epididymis (male) and perirenal tissue (Altintas et al., 2012). The leptin gene was also found in other tissues such as the brain, heart, lung, liver, kidney, stomach, and intestine (Tadokoro et al., 2015). The expression of leptin is regulated by multiple factors, such as glucocorticoids, inflammatory cytokines, and insulin (Dagogo-Jack, 2001). In addition, it has also been demonstrated that sympathetic norepinephrine release is vital to decreasing the expression of the leptin gene in response to leptin injections (Mantzoros et al., 1996). Furthermore, circulating leptin levels also affect the physiological potency of leptin. For example, hyperleptinemia show diminished responses to injected leptin, while leptin-deficient mice result in pronounced responses (Sahu, 2002).

LEP gene, which encodes leptin, is a highly conserved gene. The orthologs of leptin exist in amphibians and fish with striking differences in primary amino acid sequence. This is mainly because the key second and tertiary structures promote the formation of disulfide bridges. Leptin can bind leptin receptors in a paracrine or autocrine manner, which produces a range of central effects by influencing the hypothalamic-pituitary-target organ axis. Moreover, it can also act on many peripheral tissue cells to exert peripheral physiological effects. Therefore, leptin plays a major role in immune response, hematopoiesis, inflammation, and vascular remodeling or neovascularization.

## Leptin receptor and signaling pathway

It has been shown that leptin mediates physiological effects by acting on corresponding receptors. With advances in science and technology, the gene expression, structure, and function of leptin receptors have been investigated. The leptin receptor was previously reported located on chromosome 1p31 (Wu and Sun, 2017) The leptin receptor, a member of the cytokine receptor class I family, exists as six different isoforms (Becerril et al., 2019). These isoforms possess a common leptin binding domain instead of their intracellular domains. The leptin receptor can be distinguished in long and short isoforms (Schulz et al., 2009). In mammals, leptin regulates energy metabolism and food intake by activating the long-form leptin receptor and downstream signaling (He et al., 2012). Studies have shown that leptin is bound to the leptin receptor, which allowed the activation of JAK2 and modulated leptin receptor signal transduction (Luo et al., 2021; Costa et al., 2022). Leptin-induced PI3K signaling has important consequences for food intake and sympathetic nerve activity (Garcia-Galiano et al., 2019). Meanwhile, this signaling also induces mammalian-target-of-rapamycin (mTOR) signaling activation (Wang et al., 2012). Additionally, leptin can regulate AMPK phosphorylation, acting as a metabolic master switch in regulating energy fluxes (Xu et al., 2020). In peripheral tissues, leptin promotes fatty acid oxidation and glucose transport by stimulating AMPK activity (Koo et al., 2019; Zhang et al., 2020). Conversely, leptin inhibits the activation of AMPK in the brain, which specifically modulates food intake by regulating hypothalamic neuropeptides (Minokoshi et al., 2004). Recently, Takahashi et al. (2021) reported that JAK2/STAT3 signaling pathway was involved in wound healing by regulating angiogenesis, cell migration, and proliferation. Qing et al. (2019) demonstrated that metformin accelerated wound healing by inducing the M2 macrophage polarization by regulating the AMPK/mTOR/NLRP3 singling pathway. Li et al. (2019) reported that human amniotic mesenchymal stem cells promote wound healing by inhibiting skin cell apoptosis by activating PI3K/Akt signaling pathway. The above studies indicated that leptin may be involved in wound healing by regulating corresponding signaling pathways.

## The role of leptin in inflammation

Upon skin injury, blood floods from injured vessels into the wound area. The platelets aggregation contributes to achieving hemostasis in the wound and immune cells, including neutrophils and macrophages, concentrate in the wound area (Barman and Koh, 2020). Although many cell types play critical roles in wound healing, the macrophage has been demonstrated to be involved in the healing process, especially during inflammation stage (Boniakowski et al., 2017). Early in wound repair, macrophage leads to the production of inflammatory cytokines, clearance of debris, and production of reparative molecules (Boniakowski et al., 2017). At later stages of the inflammatory stage, the macrophage is beneficial for the resolution of inflammation and promoting tissue repair (Boniakowski et al., 2017). It has been shown that the phagocytic activity of macrophages is enhanced by leptin. Moreover, leptin knockdown significantly impaired the phagocytic activity of macrophages (Mancuso et al., 2018). In addition, leptin has also been shown to increase the expression of CD69, a marker for monocyte activation, which induces the production of even more cytokines and promotes wound healing (Santos-Alvarez et al., 1999). Goren et al. (2003a) also proved that topical administration of leptin onto wounds increased macrophage influx into the wounded areas, which suppressed inflammation and improved wound healing. This result was similar to those of previous reports (Liapakis et al., 2007; Seleit et al., 2016; Lee et al., 2018). Similarly, lymphocytes also play an important role in the inflammatory response and tissue repair. Seraphim et al. (2020) demonstrated that the lack of lymphocytes significantly impaired the wound healing conditions. Leptin has also been shown to enhance lymphocyte proliferation (Grases-Pinto et al., 2019). Therefore, leptin may mediate wound-induced inflammation by promoting the proliferation of lymphocytes. Leptin was suggested to be endowed with chemotactic activity toward neutrophils (Montecucco et al., 2006). It is a pure chemoattractant devoid of secretagogue properties but capable of inhibiting neutrophil chemotaxis to classical neutrophilic chemoattractants (Montecucco et al., 2006). Also, it was reported that leptin stimulated chemotaxis of polymorphonuclear cells and promotes T helper 1 (Th1) cell differentiation. Thus, leptin might enhance the chemotaxis of macrophages and lymphocytes, thus facilitated the wound healing process (Matarese et al., 2005). Tumor necrosis factor α (TNF-α) is considered to be a strong pro-inflammatory cytokine. Wang et al. (2020a) found that persistent treatment with TNF-α suppressed repair of the corneal epithelium after corneal epithelial debridement. Su et al. (2021) showed that leptin could inhibit the expression of TNF-α. However, whether leptin is directly involved in wound healing by affecting TNF-α remains to be further investigated.

Human b-defensin-2 (hBD-2), the small and cationic amphiphilic molecule, is known to be an endogenous mucosal antimicrobial peptide and plays a crucial role in curbing inflammation (Ghosh et al., 2018). Koeninger et al. (2020) reported that hBD-2 suppressed inflammation by decreasing the expression of NF-κB *via* binding to chemokine receptor 2. It has been reported that hBD-2 expression can be enhanced by skin wounding (Lan et al., 2012). Importantly, hBD-2 expression significantly decreased in diabetic rat skin after wounding compared with the control group (Lan et al., 2012). A further study proved that inadequate upregulation of hBD-2 might delay wound healing by inhibiting inflammation (Lan et al., 2012). The expression of hBD-2 is shown to be regulated by IL-1β (Salem et al., 2019). A study from Japan suggested that leptin dramatically improved IL-1-induced hBD-2 production (Kanda and Watanabe, 2008). Moreover, inhibition of leptin remarkably suppressed hBD-2 secretion (Kanda and Watanabe, 2008). Further study found that leptin promotes STAT1 and STAT3 activities, the hBD-2 promoter (Kanda and Watanabe, 2008). In addition, the activation of STAT1 and STAT3 were suppressed by p38 MAPK or JAK2 inhibitors (Kanda and Watanabe, 2008). Gan et al. (2014) showed that paeoniflorin upregulated hBD-2 expression in human bronchial epithelial cells *via* the p38 MAPK signaling pathway and the p38 MAPK inhibitor significantly attenuated the expression levels of hBD-2. Therefore, leptin may promote IL-1-induced hBD-2 production by activating STAT1 and STAT3 *via* p38 MAPK or JAK2. The studies mentioned above showed that leptin might inhibit inflammation by inducing hBD-2 secretion, which ameliorated wound healing.

## Leptin and angiogenesis

Angiogenesis is the creation of new blood vessels from preexisting ones and is involved in multiple physiological and pathological processes such as wound healing, mammary gland growth, tumor development, and atherosclerosis (Dangat et al., 2020; Annex and Cooke, 2021; Guillamat-Prats, 2021; Paredes et al., 2021). Angiogenesis includes multifaceted processes such as matrix degradation, endothelial cell migration and proliferation, and recruitment of mural cells (Oon et al., 2017). Insufficient blood supply has been shown to delayed wounds healing (Wang et al., 2020b). In wound healing, new capillaries appear in the wound and rapidly grow into the wound (Chakroborty et al., 2020). Meanwhile, these capillaries produce a rich vascular net to supply nutrition and oxygen to cells (Chakroborty et al., 2020). The process of angiogenesis is regulated by several endogenous growth factors and hormones including leptin, vascular endothelial growth factor (VEGF), and FGF2. It was reported that leptin was considered to be a potent angiogenic factor and its pro-angiogenic effect was affirmed using cornea pocket and chick embryo chorioallantoic membrane models (Artwohl et al., 2002). Also, another study demonstrated that leptin-induced proliferation of capillary was similar to that stimulated by VEGF, a major proangiogenic factor (Sierra-Honigmann et al., 1998). Further study found that leptin contributed to angiogenesis by increasing endothelial cell VEGF secretion (Nwadozi et al., 2019). Stallmeyer et al. (2001a) showed that systemic and topical administration of leptin-induced normal wound VEGF expressions, which improved wound healing. However, leptin failed to improve wound angiogenesis in ob/ob mice (Stallmeyer et al., 2001a). In addition, leptin significantly induced angiogenesis by improving fibroblast growth factor-2 (FGF-2) function and the application of anti-FGF-2 antibodies led to a reduction in angiogenic response to leptin, indicating that the FGF-2 signaling pathway was necessary for leptin-induced angiogenesis (Ribatti et al., 2001).

COX-2 is recognized as inducible COX isoenzymes that could catalyze the production of prostaglandins (Dorababu, 2020). Studies have suggested that COX-2 promotes angiogenesis in many cancers. COX-2 expression has been reported to be regulated by p38MAPK. Su and Wu (2020) demonstrated that miR146b-3p plays a key role in arterial thrombosis by regulating the p38-MAPK/COX-2 pathway. Another study reported that ginsenoside Rb1 prevents homocysteine-induced endothelial cell damage *via* VEGF/p38-MAPK pathway (Lan et al., 2017). Recently, Liu et al. (2021) showed that Yangyin Shengji powder significantly promotes wound healing by inhibiting inflammation *via* suppressing the COX-2/PGE2 signaling pathway. However, whether COX-2 is involved in wound healing *via* regulation of angiogenesis is still unknown. Garonna et al. (2011) reported that leptin treatment caused phosphorylation of VEGFR2 by binding leptin receptor, which led to activation of p38-MAPK, Akt, and inhibition of COX-2. Subsequently, the proliferation, directional, and migration of endothelial cells were induced (Garonna et al., 2011). Thus, it is inferred that p38-MAPK/Akt/COX-2 signaling axis is necessary for leptin’s pro-angiogenic actions and plays an important role in promoting wound healing.

Although more and more evidence have indicated that leptin affects angiogenesis. However, some held opposite opinions that leptin has no or opposing effect on angiogenesis. Since a significant elevation of leptin precedes the onset of clinical symptoms by several months, leptin, inhibiting inflammation and promoting angiogenesis, could possibly be served as a predictive marker for preeclampsia (Wang et al., 2010). Another study also found that there was an inverse correlation between leptin plasma levels and weight of prostate cancer, and high plasma leptin levels suppressed cellular proliferation and angiogenesis *in vivo* (Ribeiro et al., 2010). Furthermore, Ring et al. (2000) demonstrated that both systemic and topical leptin significantly accelerated the wound healing process through the direct action of leptin receptors. However, analyzing wound vessel density, neither systemic nor topical leptin had any significant effect on angiogenesis (Ring et al., 2000). The above-mentioned studies are contrary to other documented results regarding the association between leptin and angiogenesis (Barinaga, 1998; Umeki et al., 2014). Collectively, leptin may promote wound healing by induced angiogenesis. However, more studies are required to investigate the association between leptin and angiogenesis.

## Leptin modulates re-epithelialization and is involved in the proliferation, and differentiation of fibroblasts

The proliferation of fibroblast and keratinocytes was reported to play important roles in wound healing. Keratinocytes proliferate and migrate at the border of the wound to create a new epidermis and this process is called re-epithelialization (Jia et al., 2020). Growth factors and cytokines, potent mitogens for epithelial cells, are involved in the regulation of keratinocyte behavior and participate in re-epithelialization during skin repair. The JAK/STAT pathway is an important signaling pathway that transduces signals for growth factors and cytokines. This pathway has also been shown to be involved in wound healing by regulating cell proliferation, migration, and differentiation. The binding of a ligand to its receptor causes the phosphorylation of JAK and promotes STAT translocation to the nucleus where it targets the promoter region on DNA and activates related signaling pathways (Jere et al., 2017). The pathway is controlled by many factors including tyrosine phosphatase, receptor antagonists, and degradation of signal adaptor molecules (Jere et al., 2017). Additionally, leptin has also been proved to modulate JAK/STAT pathway. A previous study showed that JAK-2 is the only JAK activated by leptin (Kloek et al., 2002). Goren et al. (2003b) demonstrated that JAK-2 phosphorylation was observed following leptin stimulation of human and murine keratinocytes. At the same time, phosphorylation of STAT-3 was also found in human and murine keratinocytes (Goren et al., 2003b). Furthermore, leptin stimulation was necessary and sufficient to translate constitutive JAK-2 phosphorylation into a subsequent activation of STAT-3 (Goren et al., 2003b). Phosphorylation of Y1138 of leptin receptor has been showing to be the essential prerequisite for activation of STAT-3 (Gove et al., 2009). Further study found that leptin may activate STAT-3 by JAK-2-mediated phosphorylation of Y1138 of leptin receptor (Goren et al., 2003b). ERK-1 has been known to participate in wound healing by affecting keratinocyte proliferation and migration (Matsubayashi et al., 2004). Phosphorylation of ERK-1 was dramatically suppressed by treatment of its inhibitor (Yang et al., 2020). Additionally, the blockage of ERK-1 activity led to a significant reduction in wound healing (Yang et al., 2020). Stallmeyer et al. (2001b) also reported that activation of the ERK-1 pathway was involved in the mitogenic responses in primary keratinocytes. Further study found that the expression of pERK-1 significantly elevated after treating with human recombinant leptin, indicating that leptin stimulation could activate the ERK pathway in keratinocytes (Stallmeyer et al., 2001b). The above observations are consistent with previous results (Frank et al., 2000; Groschl et al., 2005; Kampfer et al., 2005; Pastar et al., 2012; Tadokoro et al., 2015). Thus, leptin may improve wound healing by enhancing the proliferation of keratinocytes through JAK/STAT or ERK pathway.

The extracellular matrix (ECM), a three-dimensional molecule network, provides structural integrity and plays a crucial role in cell survival, migration, proliferation, tissue repair, and wound healing. A study showed that fibroblasts can secrete collagen fibers which deposit at the wound to form ECM of the skin (Lu et al., 2020). It is well known that the overproduction of collagen and proteoglycans and impaired degradation of collagen structural matrix could result in excessive scar formation. During the hemostasis phase, the ECM is broken down and is replaced by matrix metalloproteases produced by fibroblasts (den Dekker et al., 2019). At the same time, ECM proteins add to the granulation tissue (den Dekker et al., 2019). Fibroblasts maintain the ECM in uninjured tissue and are summoned to the wound site by chemoattractants, such as IL-1β, TNF-α, and platelet-derived growth factor (PDGF) (Bainbridge, 2013). Also, fibroblasts have been proved to contain leptin receptors and secrete various matrix metalloproteinases. Williams et al. (2016) confirmed that leptin increased the expression and activity of matrix metalloproteinase-1, -3, -8, and -14 in human gingival fibroblasts. Whether leptin is involved in wound healing *via* modulating fibroblasts is not revealed. Recently, Seleit et al. (2016) reported that a significant positive correlation was observed between a higher leptin H score and positive family history in keloid. Further study found that leptin overexpression may participate in keloid formation by altering cytokine production, prolonging healing phases with excessive deposition, and delaying collagen degradation (Seleit et al., 2016). In addition, leptin not only directly stimulates the synthesis of procollagen, fibronectin, laminin, and glycosaminoglycans from proliferating fibroblasts, but also significantly increased the expression level of tissue inhibitors of matrix metalloproteinase, blocking collagen degradation (Kizu et al., 1996; Schulz and Widmaier, 2004). Hydrocellular foam dressings (HCF), a type of wound dressings, are made of a three-layer structure. HCF is reported to promote wound healing by absorbing excessive wound fluid containing various cytokines and growth factors. Importantly, Yoshino et al. (Yoshino et al., 2015) demonstrated that HCF significantly increased leptin levels and upregulated fibroblast proliferation in the wound fluid. However, leptin antagonists could block fibroblast proliferation induced by HCF (Yoshino et al., 2015). Meanwhile, leptin antagonists dramatically suppressed HCF-induced wound healing (Yoshino et al., 2015). This suggests that HCF may improve wound healing through leptin-mediated fibroblast proliferation. The aforementioned studies showed that leptin can lead to an increased scar formation by promoting the collagen-secreting ability of fibroblast or ameliorating wound healing by enhancing fibroblast proliferation.

**FIGURE 1:**
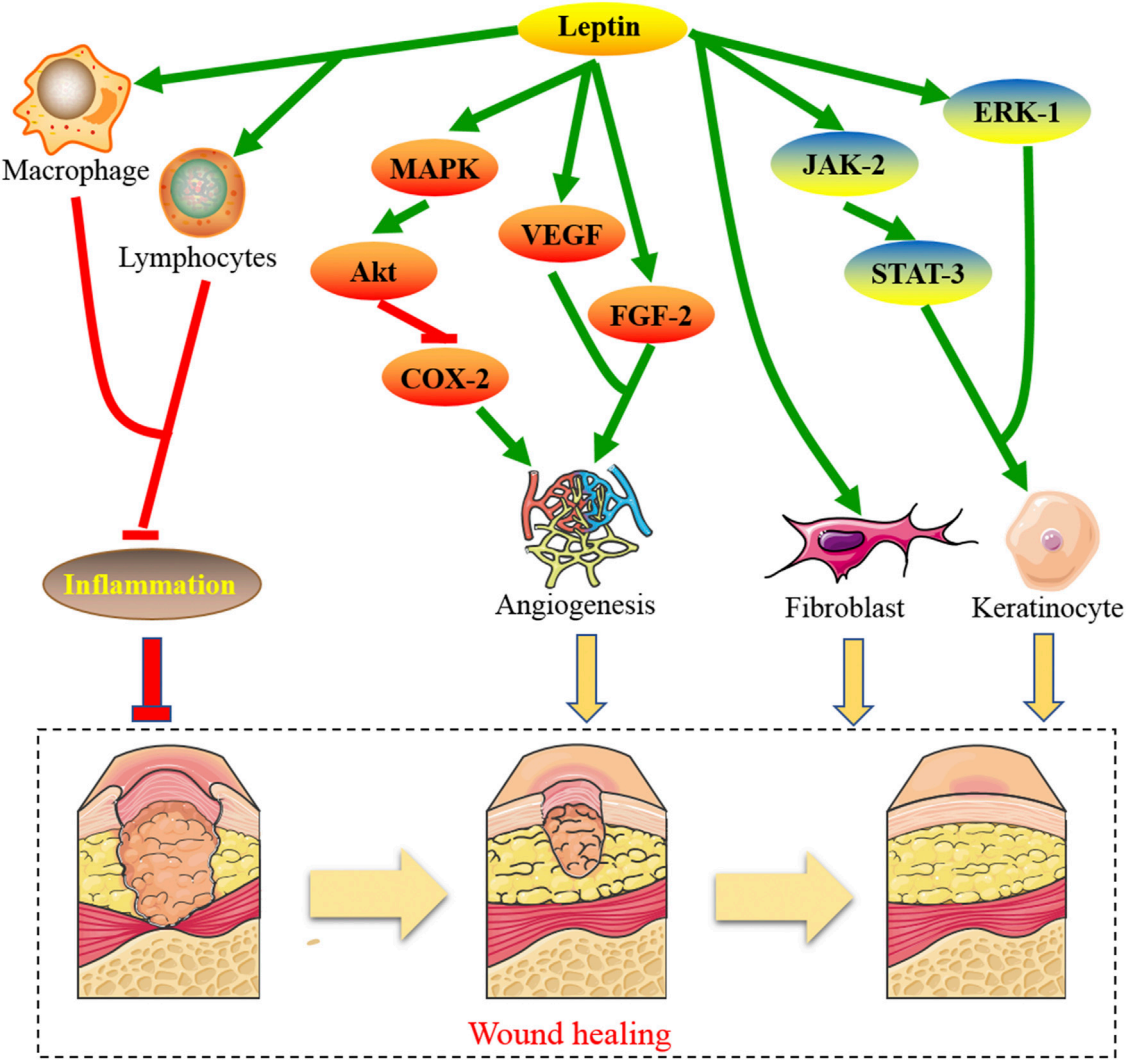
The molecular mechanisms by which leptin contributes to the treatment of wound healing.

**FIGURE 2:**
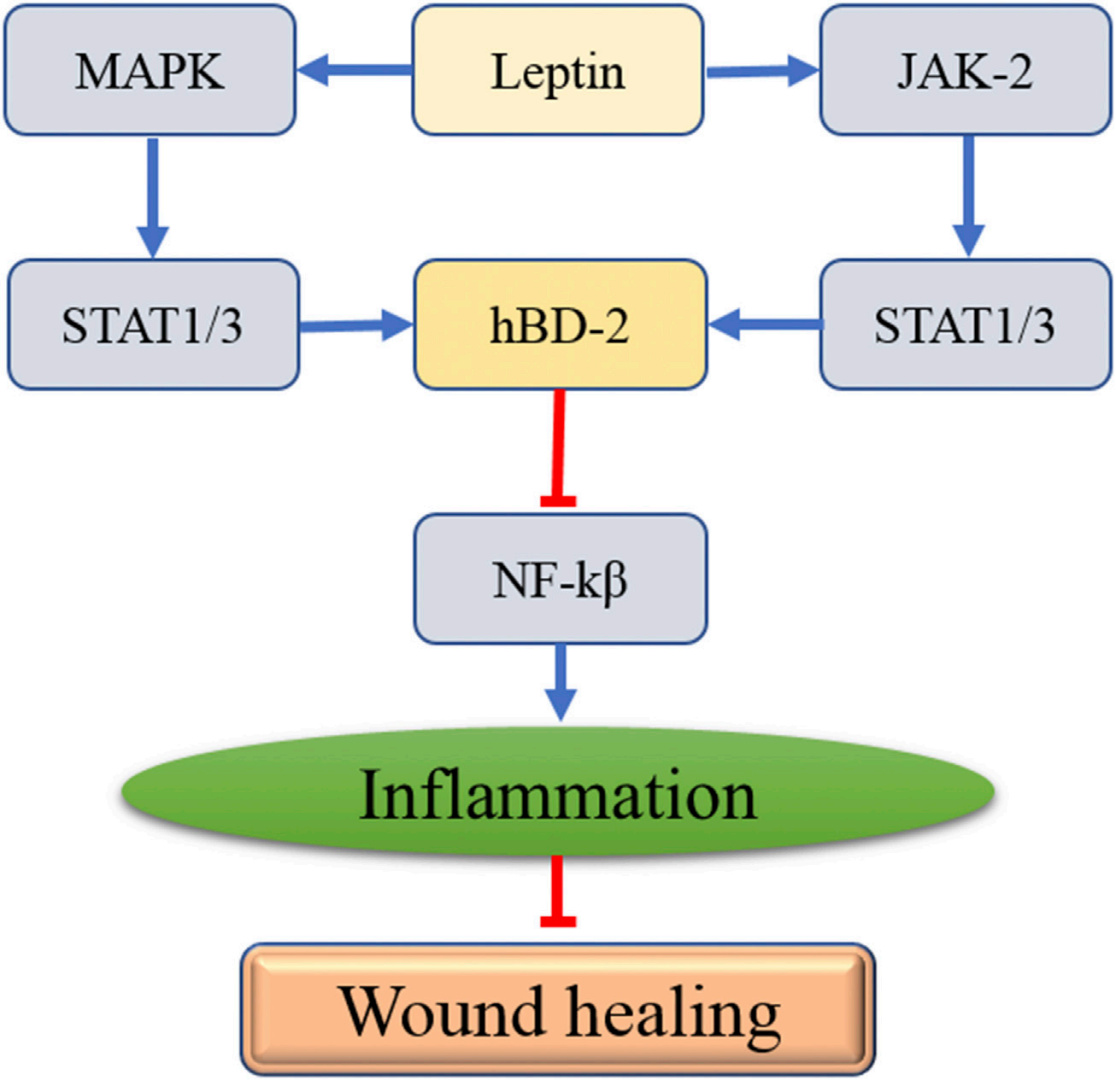
The interaction between leptin and the affected genes in the process of wound healing.

**FIGURE 3:**
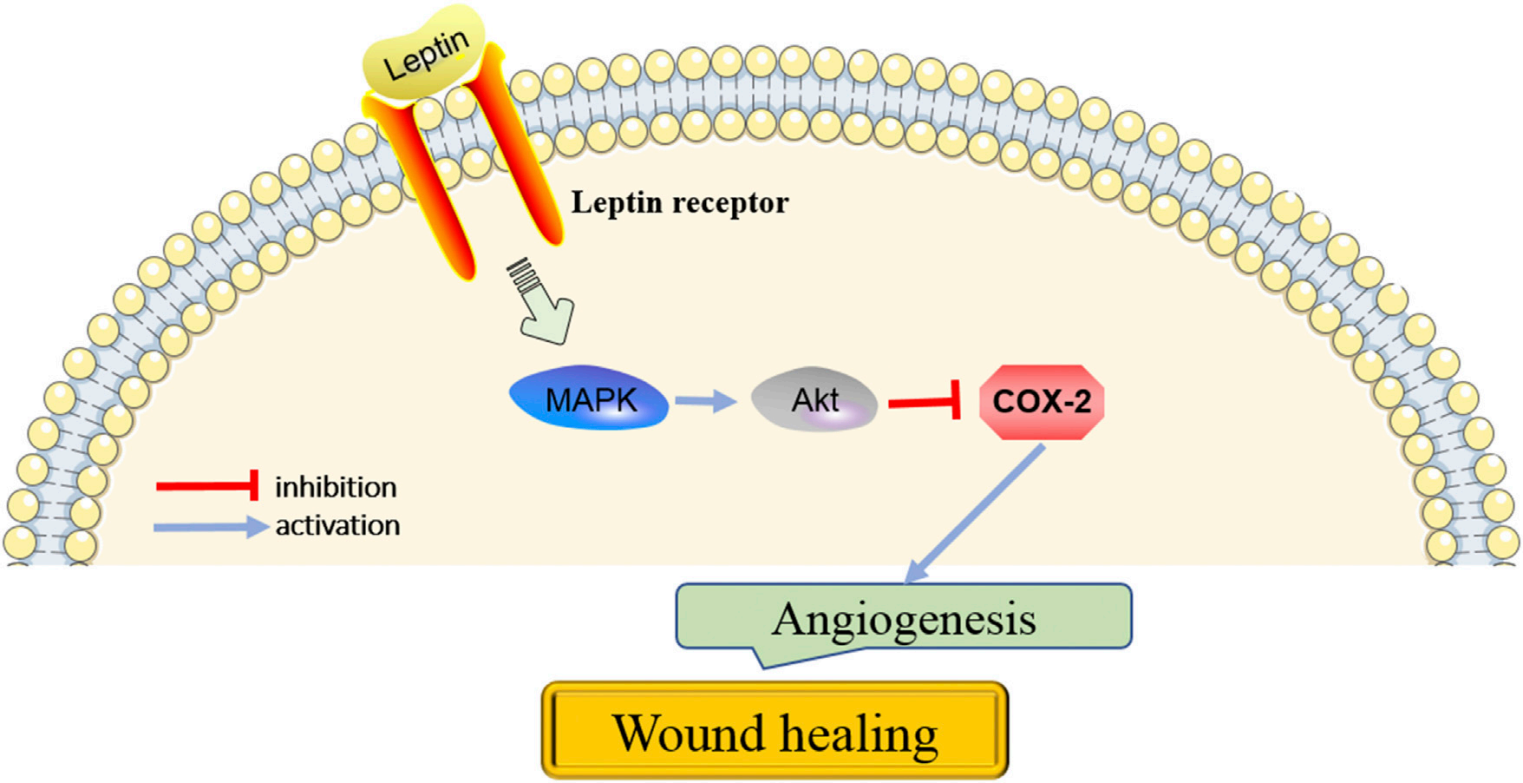
The key pathways in the action of leptin on angiogenesis for wound healing.

Figures1–3, showed the molecular mechanisms by which leptin and the associated genes contributed to the treatment of wound healing.

## Directions for future researches

Wound-healing disorders are a therapeutic problem of extensive clinical importance. Based on the above evidence from *in vitro* and *in vivo* studies, leptin is one of the key factors on the promotion of wound healing in the skin, which may be associated with its involvement in multiple biological process of wound healing, including the acceleration of proliferation, differentiation of the epidermal keratinocytes, and enhancement of angiogenesis around the wounded area. Therefore, leptin might serve as an attractive potential therapy for wound-healing disorders. However, few studies have reported the exact effects of leptin on wound-healing illnesses in clinical practice presently. Future clinical trials are warranted to validate this multifunctional and potent systemic hormone on wound healing in the human skin, which may pave the way for the clinical utilization of leptin as a wound healing-promoting agent.

## Conclusion

Leptin, an adipocyte-derived hormone, mainly acts in the hypothalamus and plays crucial roles in distinct biological processes, such as energy expenditure and wound healing. Although some previous studies showed that leptin played an important role in wound healing, the understanding of the specific role in each process of wound repair is not entirely clear, and further researches are needed on both macrostructural and microscale factors. Wound healing is a messy and orderly process and many tissues, organs, cells, and cytokines are involved in this process. Therefore, identifying and validating the mechanism of leptin regulating inflammation, angiogenesis and re-epithelialization are of great significance to develop potential therapeutic targets for the treatment of wound healing in clinical practice.
